# Estimation of Organizational Competitiveness by a Hybrid of One-Dimensional Convolutional Neural Networks and Self-Organizing Maps Using Physiological Signals for Emotional Analysis of Employees

**DOI:** 10.3390/s21113760

**Published:** 2021-05-28

**Authors:** Saad Awadh Alanazi, Madallah Alruwaili, Fahad Ahmad, Alaa Alaerjan, Nasser Alshammari

**Affiliations:** 1 Department of Computer Science, College of Computer and Information Sciences, Jouf University, Sakaka 72341, Saudi Arabia; asalaerjan@ju.edu.sa (A.A.); nashamri@ju.edu.sa (N.A.); 2 Department of Computer Engineering and Networks, College of Computer and Information Sciences, Jouf University, Sakaka 72341, Saudi Arabia; madallah@ju.edu.sa; 3 Department of Basic Sciences, Deanship of Common First Year, Jouf University, Sakaka 72341, Saudi Arabia; drfahadahmadmian@gmail.com

**Keywords:** emotional intelligence, physiological signals, blood volume pulse, galvanic skin response, skin temperature, organizational competitiveness, one-dimensional convolutional neural network, self-organizing maps

## Abstract

The theory of modern organizations considers emotional intelligence to be the metric for tools that enable organizations to create a competitive vision. It also helps corporate leaders enthusiastically adhere to the vision and energize organizational stakeholders to accomplish the vision. In this study, the one-dimensional convolutional neural network classification model is initially employed to interpret and evaluate shifts in emotion over a period by categorizing emotional states that occur at particular moments during mutual interaction using physiological signals. The self-organizing map technique is implemented to cluster overall organizational emotions to represent organizational competitiveness. The analysis of variance test results indicates no significant difference in age and body mass index for participants exhibiting different emotions. However, a significant mean difference was observed for the blood volume pulse, galvanic skin response, skin temperature, valence, and arousal values, indicating the effectiveness of the chosen physiological sensors and their measures to analyze emotions for organizational competitiveness. We achieved 99.8% classification accuracy for emotions using the proposed technique. The study precisely identifies the emotions and locates a connection between emotional intelligence and organizational competitiveness (i.e., a positive relationship with employees augments organizational competitiveness).

## 1. Introduction

Organizational success is highly dependent on the competitiveness of the workforce, products, and services in rapidly changing market conditions. As a result, enhancing enterprise competitiveness is a popular topic in today’s competitive business environment. Existing research demonstrates that strengthening leadership and overall organizational behavior, establishing positive relationships with partners, upgrading technology, and making capital investments effectively contribute to organizational competitiveness [1,2].

Human capital has emerged as the primary driver of economic growth and industrial development in modern economies. Corporate technological creativity has also developed into a critical component of enterprise development. People are an organization’s primary resource because they are instrumental in sustaining its competitive advantage and enhancing performance. Thus, organizations should prioritize providing a healthy work environment with all the necessary facilities. Once organizations provide a comfortable working environment, employee satisfaction results in more productivity [1].

Employees develop a stronger connection to their organizations due to the internal link between emotions and the work environment, culminating in more active and positive attitudes toward the organization. As a direct consequence of these combined factors, organizational competitiveness improves [2].

Emotional computing (EC) should be considered because it is a broad term that encompasses the study of emotional analysis [2,3]. Emotion detection using physiological data is an escalating field of research that has produced numerous studies examining various emotions and analysis approaches. Following the rapid adoption of smart technologies in society, the human emotion recognition system has received considerable attention. Additionally, emotion recognition is a highly effective technique for assessing human emotional states and forecasting behavior to improve performance in various fields. Organizational competitiveness and employee relations are no exception. In recent years, researchers have frequently used emotional stimuli, such as images, sounds, and videos, to elicit subjects’ emotions and analyze their physiological signals to determine the regularity of emotional changes [4,5]. Human emotion recognition is accomplished through either a face recognition system or sensor-based systems.

Regarding the face recognition system, it is simple to smile and attempt to express joy, which means that the system is easily deceived and cannot be relied upon as a reliable source of information. In contrast, sensor-based systems that rely on physiological signals are significantly more challenging to deceive. Additionally, physiological signals fluctuate rapidly in response to changing emotions [6] and are spontaneous reactions that cannot conceal emotions [7]. These signals are more challenging to manipulate than facial expressions or strained vocalizations [8]. Therefore, the importance of a sensor-based emotion recognition system has increased because it provides a more objective method of measuring emotions. Sensors in emotion recognition systems are used to communicate subliminally with a person. The sensors receive feedback from a person regarding something the person feels, sees, or hears [2,9,10,11,12].

Among a variety of physiological signals, previous research has established that the photoplethysmogram (PPG), galvanic skin response (GSR), and skin temperature (SKT) provide the most indicative methods for assessing emotion. Thus, we chose to acquire and analyze only the PPG, GSR, and SKT signals because appropriate wearable devices can collect signals from a person without jeopardizing comfort or privacy. Only two electrodes on the nondominant hand are necessary to acquire the PPG, GSR, and SKT signals. Other signals lack this ease of use. For example, the subject must wear a headset or helmet to collect electroencephalogram (EEG) signals, and the subject must place electrodes on his or her chest to collect electrocardiogram (ECG) signals [8].

The advent of sensors and wearable devices as mechanisms for acquiring physiological data from people in their daily lives has enabled research into emotional pattern recognition to identify user emotions in various contexts [13]. The PPG signal detects changes in the blood volume in the tissue using a pulse oximeter. Typically, a PPG signal is extracted from the finger, and numerous electrical devices incorporate PPG sensor functions. Another advantage of the PPG signal is that it is dynamic. As a result, several researchers have employed PPG signals for emotion recognition [14,15].

The GSR is a noninvasive method for continuously measuring the electrical parameters of human skin. Skin conductance is frequently used as the primary parameter in this technique. The electrical parameters of the skin are not under conscious human control. These parameters are dependent on the variation of the sweat reaction, which reflects changes in the sympathetic nervous system, according to traditional theory [8]. There is evidence that changes follow specific sympathetic nervous burst output signals in skin conductance. Emotional changes cause sweating, which is most noticeable on the palms, fingers, and soles. The sweat reaction alters the amount of salt on the skin, which results in a change in the skin’s electrical resistance [16]. Emotions have an instantaneous effect on the human brain, boosting blood flow to the skin and altering the skin temperature measured by the SKT sensor.

**Figure 1:**
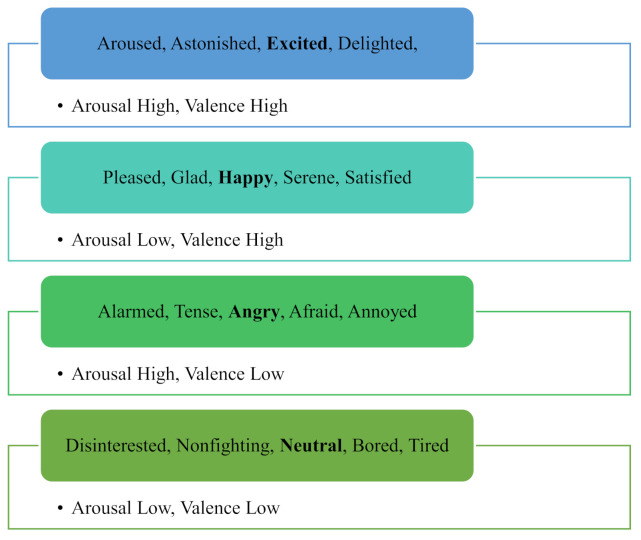
Circumplex model presenting arousal and valence in different emotional states adapted from [21].

Emotional categories are defined in a circular structural model that includes basic emotions (e.g., excited, happy, pleased, relaxed, peaceful, calm, sleepy, bored, sad, nervous, angry, and annoyed) to define the arousal and valence dimensions. Typically, two emotional dimensions from Russell’s valence–arousal emotion model are used to evaluate emotions in the study of emotion recognition [17,18]. The majority of studies employ variants of Russel’s circumplex model of emotions, as illustrated in Figure 1, which depicts the valence and arousal distributions of basic emotions [19,20].

The research was conducted to determine the feasibility of automatically recognizing arousal and valence levels in 25 healthy subjects triggered with sets of affective sounds from the International Affective Digitized Sound system database. Stimuli were quantified using the circumplex affect model, and electrodermal activity (EDA) was analyzed. Three levels of arousal stimuli were used in the experiment, each containing pleasant and unpleasant sounds. The statistical analysis revealed no significant differences in the neurogenic feature set between the three arousal groups of stimuli and the two valence levels. Instead, the mean tonic value varied significantly across the three arousal levels and two valence levels. The classification procedure was conducted on four distinct datasets to distinguish between the two valence levels and three arousal levels using only positive sounds, only negative sounds, and both positive and negative stimuli without discrimination. The results from the convex optimization approach to electrodermal activity indicated an 80.00% recognition accuracy on the arousal dimension. With respect to only positive and negative sounds, the arousal identification results confirmed a high discrimination power of more than 77%, demonstrating the minimal effect of the valence dimension on the arousal classification. Additionally, excellent performance was obtained in the valence classification with 84% accuracy overall [22].

As an artificial intelligence method, deep learning has been widely employed in emotion analysis, image recognition, video segmentation, speech, and neural language programming. Artificial intelligence has developed rapidly and has attracted greater attention in academia and industry [23].

Artificial neural networks (ANNs) provide an effective solution to various cognitive tasks by simulating human learning behavior. The one-dimensional convolutional neural network (ODCNN) and the self-organizing map (SOM) have been recognized as the most effective ANN models. As a standard deep learning method, the ODCNN has several advantages: it can easily extract features from the original dataset without manual selection and adjust and optimize convolutional kernel parameters during training to achieve dataset classification and recognition. The CNN is based on the principle of learning patterns and correlations from numerous data points. If the data point is numeric, it is critical to note that data points must display real-world information. The effectiveness of the SOM in clustering has been widely recognized; thus, it can be used to discover high-level cognitive operations, such as emotions. The SOM has been used to visualize relationships between classified objects within a cluster and between different clusters within a dataset [24,25].

According to previous research, worker productivity is critical for any company or organization seeking to increase performance, lower costs, maximize revenue, and increase competitiveness. Productivity analysis is a frequently studied topic in business management. Earlier research has examined worker productivity through the perspective of job-related concerns [26,27,28], technological innovation [29,30], employee demographics and socioeconomic characteristics [31,32], and conditions affecting employee mental and physical health [33,34]. Although prior studies have investigated various factors affecting worker productivity, several studies have examined the relationship between workers’ on-the-job emotional states and organizational performance [35,36]. This paper evaluates machine learning and deep learning tools to determine organizational competitiveness using employee physiological signals at the workplace for emotional analysis.

The paper is organized as follows. Section 2 explains the materials and methods, and the experiments and results are analyzed and discussed in Section 3. Section 4 presents the comparative analysis. Finally, the conclusion and future work are presented in Section 5.

## 2. Materials and Methods

### 2.1. Dataset for the Proposed Work

Regarding the data collection (i.e., measurement), three strategies are used to classify emotions: (i) neurological/physiological measurement using sensors to detect changes in the user’s body; (ii) subjective self-reporting using questionnaires, diaries, or interviews; and (iii) behavioral measurement based on expert observations of the participant’s behavior. While all these approaches have their specific advantages and disadvantages, as Kim and Fesenmaier suggested, physiological measurement is particularly objective [37]. In this manuscript, different repositories [38,39,40,41] are used for the input data acquisition. These datasets facilitate generating data for this research. They contain organizational ranking (1–10), subject ID (referring to the person), gender, age, height, weight, body mass index (BMI), blood volume pulse (BVP), GSR, SKT, arousal, and valence and the respective emotional state, which helps categorize organizational competitiveness (i.e., highly competitive, moderately competitive, or less competitive). A value of 1 to 5 for organizational rank depicts a high level of organizational competitiveness, and a value of 6 to 8 indicates a moderate level of organizational competitiveness. Finally, a value of 9 to 10 represents a low level of organizational competitiveness.

### 2.2. Emotion Classification through the Convolutional Neural Network

The traditional CNN is intended to work solely on two-dimensional (2D) datasets, such as videos and images. Thus, they are regularly called 2D CNNs (TDCNNs). Another option, a variant of the TDCNN, the ODCNN, is employed in this research for physiological signal-oriented emotional state detection (using a preprocessed/numeric dataset). For specific usage, ODCNNs are beneficial and desirable over their 2D counterparts in managing a one-dimensional (1D) dataset for the following reasons:

- Instead of matrix operations, basic array operations are required for forward and backward propagation in the ODCNN, which implies that the computational complexity of the ODCNN is less than that of the TDCNN.
- Recent investigations have indicated that the ODCNN with generally shallow designs (e.g., fewer neurons and hidden layers) can perform learning (for taxing operations), including 1D input data.
- Typically, a TDCNN demands additional deep models to deal with such cases. Networks with shallow structures are much simpler for training and execution.
- Usually, preparing deeper TDCNNs demands a particular setup for hardware. However, the feasibility of any processor execution over a standard computer, moderately quick for preparing the ODCNN with some neurons (e.g., fewer than 50) and hidden layers (e.g., two or fewer), is straightforward.
- Fewer computational necessities make the ODCNN appropriate for low-cost, real-time, and well-suited applications, particularly on portable or hand-held gadgets.

**Figure 2:**
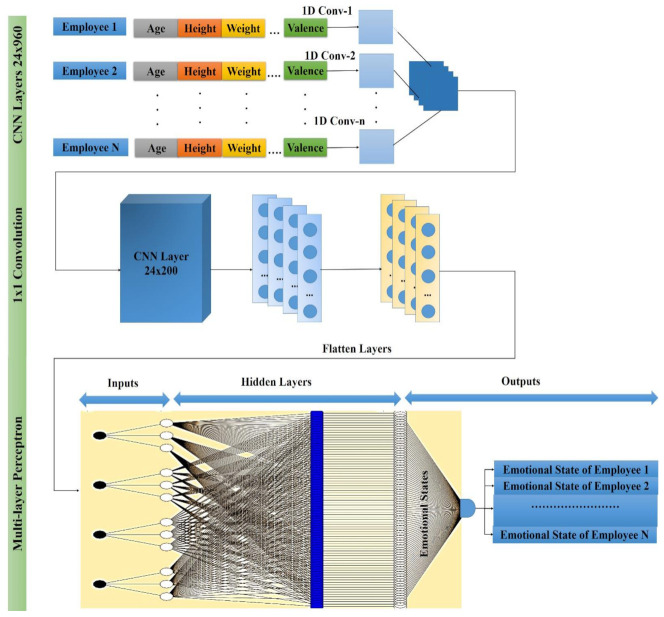
Proposed model for emotional state detection based on the one-dimensional convolutional neural network (ODCNN).

The ODCNN has remarkable execution on numerous other applications with a high signal variety and very few labeled data gained through various means. This research aims to use this preprocessed dataset obtained through physiological signals for emotional state detection. The presented model in Figure 2 has the following layers: two layers in the ODCNN (i.e., the typical layers of the CNN: 1D convolutions and the pooling layer) and fully connected layers that are identical to the multilayer perceptron (MLP).

The accompanying hyperparameters shape the ODCNN’s configuration:

- Hidden layers of the MLP and hidden layers of the CNN are used.
- In every layer of the CNN, the kernel/filter size is selected.
- In each CNN layer, a subsampling factor is selected.
- The activation and pooling functions are selected.

The *l*th neuron in the hidden layer of the CNN (i.e., *m*) carries out the convolutional sequence. Following this, the subsampling operation (i.e., the actuation work, *f*) is used for its summation. It is the primary contrast between the ODCNN and TDCNN. For feature maps and kernels, the 2D matrix is replaced with a 1D array. As a subsequent stage, the 1D input data (i.e., physiological data) are processed by the CNN layers, and it determines how to perform the extraction (through MLP layers) of such features used in the detection and classification of emotions. As an outcome, feature extraction and classification are optimized by fusing them into a process. The ODCNN has low computational complexity (as the single function with high cost is due to a series of 1D convolutions that comprise two 1D arrays). During backward and forward propagation, parallel execution of linear functions is performed. This method is adaptive because it permits the changes in the dimensions of the input layer to adaptively tune the subsampling factor of the output layer of the CNN (for emotional state classification).

#### Backward and Forward Propagation in the One-Dimensional Convolutional Neural Network

The mathematical expression for forward propagation in every layer of the ODCNN is given below (Equation (1)):

(1) $$y_{l}^{m}=C_{l}^{m}+\sum_{j=1}^{O_{m-1}}conv1D\;(x_{jl}^{m-1},\;t_{j}^{m-1}),$$

where $y_{l}^{m}$ is characterized as the input parameters (i.e., age, height, weight, BMI, BVP, GSR, SKT, valence, and arousal). At the ***m***th layer, $C_{l}^{m}$ is characterized as a bias of the *l*th neuron. At the (***m*** − 1)th layer, $t_{j}^{m-1}$ is the outcome of the ***j***th neuron, and $x_{jl}^{m-1}$ is the filter from the ***j***th neuron (at the (***m*** − 1)th layer) to the ***l***th neuron (at the *m*th layer). Without zero padding, ***conv*-1D** (., .) is used to carry out legitimate 1D convolutions. Subsequently, the input array dimension $y_{l}^{m}$ is less than the output array dimension (i.e., $t_{j}^{m-1}$). With the passage of input parameters (i.e., $y_{l}^{m}$) from the activation function *f*(.), the intermediate result $z_{l}^{m}$ is presented below (Equation (2)):

(2) $$z_{l}^{m}=f\;(y_{l}^{m})\;\mathrm{and}\;t_{l}^{m}=z_{l}^{m}\;↓\;\mathrm{tt},$$

where $t_{l}^{m}$ represents the ***l***th neuron’s outcome at the ***m***th layer, and the down-sampling operation is addressed by “↓ tt” with a scalar factor (i.e., tt). The backpropagation scheme can be summed as given below. From the output layer of the MLP, the backpropagation scheme for the error starts. Suppose that ***m*** = 1 for the input layer and ***m*** = **M** for the output layer. Additionally, suppose that **OM** is the number of classes in the dataset (four classes of emotional states: neutral, happy, excited, and angry). Thus, for an input vector q, the output and target vector u^q^ is [$z_{1}^{m}$, ⋯, $z_{Om}^{m}$]′.

For the q input parameters in the output layer, the mean squared error (MSE), ***F_q_***, is expressed below (Equation (3)):

(3) $$F_{q}=\mathrm{MSE}\;(u^{q},\;[z_{1}^{m},\;\cdots ,\;z_{Om}^{m}]\prime )=\sum_{j=1}^{O_{m}}{\left(z_{j}^{m}-\;u_{j}^{q}\right)}^{2}.$$

The delta error, $\Delta_{l}^{m}$ = ***∂F* *∂ylm***, is calculated through every network parameter to determine the derivative of ***F_q_***. In particular, the chain rule of derivatives can be employed (in the previous layer) to refresh the weights and bias of the neurons (Equation (4)).

(4) $$\frac{\partial F}{\partial x_{ml}^{m-1}}=\Delta_{l}^{m}z_{j}^{m-1}\;\mathrm{and}\;\frac{\partial F}{\partial c_{l}^{m}}=\Delta_{l}^{m}.$$

The backpropagation procedure from the first layer of the CNN to the last layer of the MLP is given below (Equation (5)):

(5) $$\frac{\partial F}{\partial t_{l}^{m}}=\Delta t_{l}^{m}=\sum_{J=1}^{O_{m+1}}\frac{\partial F}{\partial y_{j}^{m+1}}\frac{\partial y_{j}^{m+1}}{\partial t_{l}^{m}}=\sum_{J=1}^{O_{m+1}}\Delta_{j}^{m+1}\;x_{lj}^{m}.$$

When the first backward propagation is conducted from the (***m*** + 1)th to the ***m***th layer, backpropagation is performed on the input delta for the ***m***th layer of the CNN. Assuming the zero-order up-examined map is $vt_{l}^{m}\;$= vq($t_{l}^{m}$), the delta error can be communicated as presented below (Equation (6)):

(6) $$\Delta_{l}^{m}=\frac{\partial F}{\partial z_{l}^{m}}\frac{\partial z_{l}^{m}}{\partial y_{l}^{m}}=\frac{\partial F}{\partial vt_{l}^{m}}\frac{\partial vt_{l}^{m}}{\partial z_{l}^{m}}\;f^{\prime }\;(y_{l}^{m})=\mathrm{vq}(\Delta t_{l}^{m})\;f^{\prime }\;(y_{l}^{m}),$$

where $\gamma ={\left(tt\right)}^{-1}$. The backward propagation of the delta error ($\Delta t_{l}^{m}\overset{\sum \;}{\leftarrow }\Delta_{j}^{m+1}$) is given below (Equation (7)):

(7) $$\Delta t_{l}^{m}=\sum_{j=1}^{O_{m+1}}conv1Da(\Delta_{j}^{m+1},\;rev(x_{lj}^{m})).$$

The calculation ***rev*** (.) is used to reverse the array, and ***conv*1*D*a** (., .) is employed to conduct zero-padding-oriented 1D convolutions. The bias and weight sensitivities are presented below (Equation (8)):

(8) $$\frac{\partial F}{\partial x_{jl}^{m}}=convOD(t_{l}^{m},\;\Delta_{j}^{m+1})\;\mathrm{and}\;\frac{\partial F}{\partial c_{l}^{m}}=\sum_{o}\Delta_{l}^{m}\;(o).$$

The bias and weight sensitivities are calculated to refresh the weights and biases with the learning factor ***ε***, as given below (Equation (9)):

(9) $$x_{jl}^{m-1}(u+1)=x_{jl}^{m-1}(u)-\varepsilon \frac{\partial F}{\partial x_{jl}^{m-1}}\;and\;c_{l}^{m}(u+1)=c_{l}^{m}(u)-\varepsilon \frac{\partial F}{\partial c_{l}^{m}}.$$

**Figure 3:**
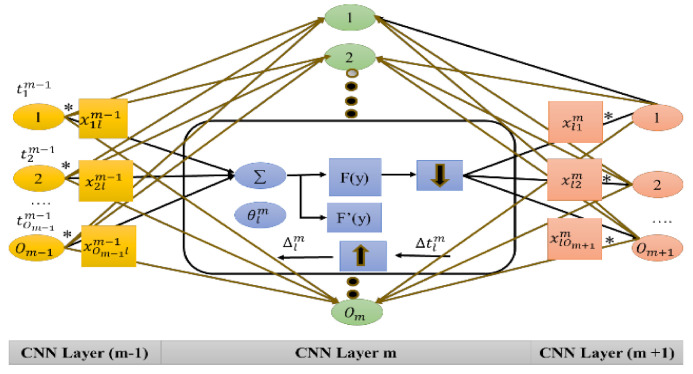
Backward and forward propagation in the one-dimensional convolutional neural network (ODCNN). Where * represents the product function during forward and backward propagation procedures.

For the ***m***th layer of the CNN, at the ***l***th neuron, $\Delta t_{l}^{m}$ is framed by backpropagating the entire delta error (i.e., $\Delta_{j}^{m+1}$) using Equation (7) at the following layer, ***m***+. The iterative backward and forward progressions for the physiological signals in the dataset are expressed in Figure 3 and explained step by step through Algorithm 1.
**Algorithm 1.** Backward and forward propagation in the one-dimensional convolutional neural network (ODCNN)
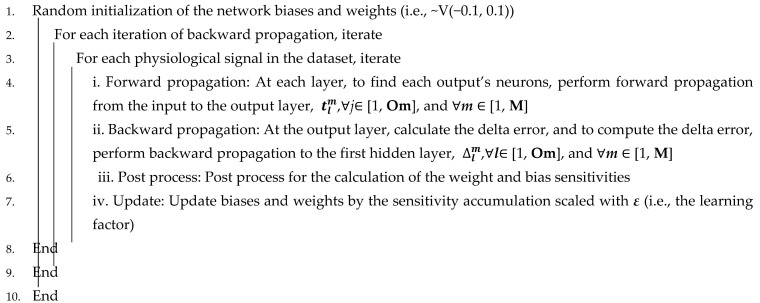


### 2.3. Detection of Organizational Competitiveness Based on the Self-Organizing Map

Due to extensive learning rather than error amendment learning, the SOM exhibits robust outcomes for the weight modification. Just the single node is used at every cycle where the neural network is provided with the parameters of the input vector’s instance (entire nodes try for the best reaction to the input).

The best matching unit (BMU)—the selected node—is chosen according to the likeness among all grid nodes and the present input values (emotional state). The smallest Euclidean distance-based node and the input vector are chosen (inside a specific radius) along the nodes (adjoining) to have their position marginally managed to coordinate with the input vector.

After traversing each node of the grid, the whole grid is matched with the input data (i.e., the emotional state of the employee), where identical nodes are grouped, and the distinct nodes are isolated. This grouping is performed to cluster the emotional state of each employee to categorize the organization’s overall competitiveness (highly competitive, moderately competitive, or less competitive).

**Figure 4:**
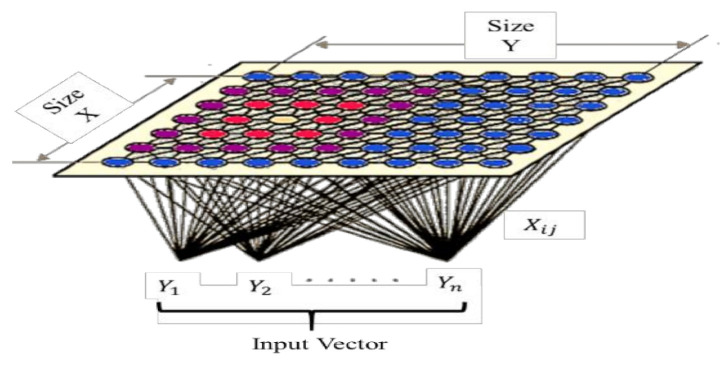
Structural representation of a self-organizing map.

**Figure 5:**
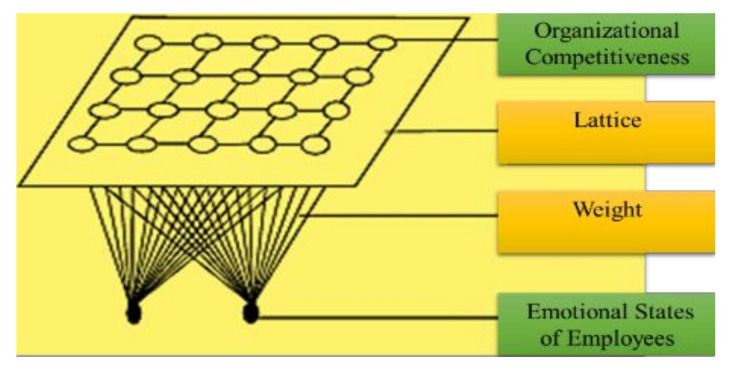
Self-organizing map with input and output labels.

Figure 4 represents the SOM structure where the outside nodes are represented by blue, the inner neighborhood radius is represented by purple and pink, and yellow represents the BMU. The parameters are presented below:

- The *U* represents the current iteration.
- The total number of iterations of the network is represented by *o*, the limit of iterations.
- For the decay of the learning rate and radius, the time constraint is represented by *λ*.
- The row coordinate of the node matrix is represented by *j*.
- The column coordinate of the node matrix is represented by *k*.
- The distance between the BMU and node is represented by *e*.
- The weight vector is represented by *x*.
- The connection weight between the input vector occurrence and *i*th and *j*th nodes in the matrix (for the *t*th iteration) is represented by ***x*_*ij***(***t***).
- The input vector is represented by *y*.
- The instance of the input vector at the *t*th iteration is represented by *y*(*t*).
- The learning rate is represented by ***β***(*t*), in the [0,1] range. To guarantee the coverage of the network, it diminishes with time.
- The neighborhood operation is represented by γ **_*ij***(***t***), which diminishes and addresses the distance between the BMU and the *i* and *j* nodes and its effect on the learning rate at the *t*th iteration.
- The neighborhood operations radius is represented by α (*t*), which determines (in the 2D matrix) how far neighbor nodes are inspected with the update of the vectors. It is progressively diminished after some time. Figure 5 presents the SOM with the input and output identification.

#### Update of Parameters in the Self-Organizing Map

The accompanying mathematical expressions perform the parameter updates. The neighborhood weight update is performed as presented below (Equations (10) and (11)):

(10) $$x{\_}_{ij}(t+1)=x_{ij}\;(t)+\beta_{i}\;(t)[y(t)-x_{ij}\left(t\right)],$$

or

(11) $$x{\_}_{ij}(t+1)=x_{ij}\;(t)+\beta_{i}\;(t)\;\gamma_{ij}\;(t)\;[y(t)-x_{ij}\left(t\right)].$$

Equation (10) reveals that the newly refreshed weight ***x*_*ij***(***t*** + 1) for the *i* and *j* nodes is equivalent to the summation of the prior weights ***x*_*ij***(***t***) and a ratio of the distinction between the input vector *y*(***t***) and the prior weights. The weight vector is transferred near the input vector. Another significant component is the weight corresponding to the 2D distance between the BMU and the nodes in the neighborhood radius.

Moreover, it does not represent the learning influence (relative to the distance between the BMU and the node). As the measure of learning should diminish according to distance, the updated weight must be well thought out, considering that the learning results are nearly none at the neighborhood limits. Accordingly, the following condition adds the additional γ_***ij***(***t***) neighborhood operation and is the most precise.

**Figure 6:**
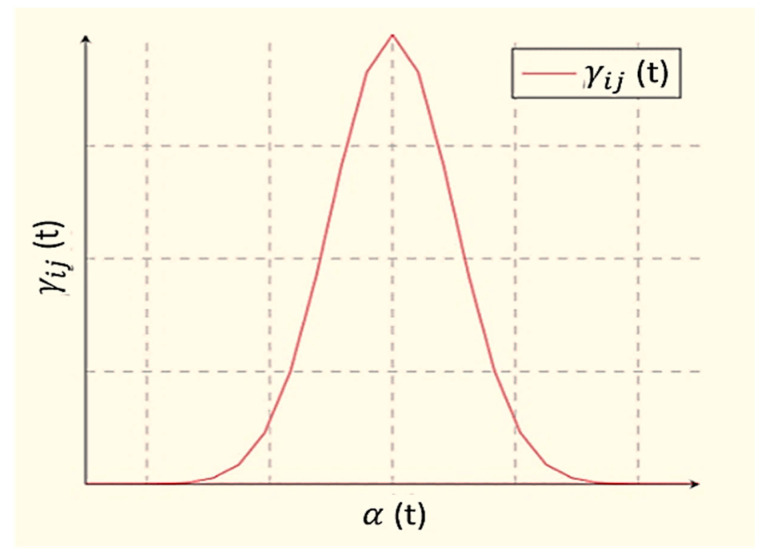
Relation between the learning rate and radius.

Figure 6 indicates that the learning rate and radius both decay exponentially (Equations (12) and (13)):

(12) $$\alpha \left(t\right)=\alpha_{0}(t)\;.\;\exp\;(\frac{-t}{\lambda }),\;\mathrm{where}\;t=1,\;2,\;3,\;\ldots n$$

(13) $$\beta \left(t\right)=\beta_{0}(t)\;.\;\exp\;(\frac{-t}{\lambda }),\;\mathrm{where}\;t=1,\;2,\;3,\;\ldots n$$

The neighborhood operation can be computed as shown below (Equation (14)):

(14) $$\gamma_{ij}\left(t\right)=\exp\;(\frac{-e^{2}}{2\;\alpha^{2}\left(t\right)}),\;\mathrm{where}\;t=1,\;2,\;3,\;\ldots n.$$

The Pythagorean theorem is used for the Euclidean distance calculation (Equation (15)):

(15) $$\left\|\begin{array}{cc} \vec{y} & \vec{x_{ij}} \end{array}\right\|=\sqrt{\sum_{t=0}^{n}{\left[\begin{array}{cc} \vec{y}\;\left(t\right) & \vec{x_{ij}\;} \end{array}\;\left(t\right)\right]}^{2}.}$$

From the computed distances of each node, the BMU is selected with the least distance (Equation (16)).

(16) $$e=\min\;(\|\begin{array}{cc} \vec{y} & \vec{x_{ij}} \end{array}\|)=\min\;(\sqrt{\sum_{t=0}^{n}{\left[\begin{array}{cc} \vec{y}\;\left(t\right) & \vec{x_{ij}\;} \end{array}\;\left(t\right)\right]}^{2}}).$$

**Figure 7:**
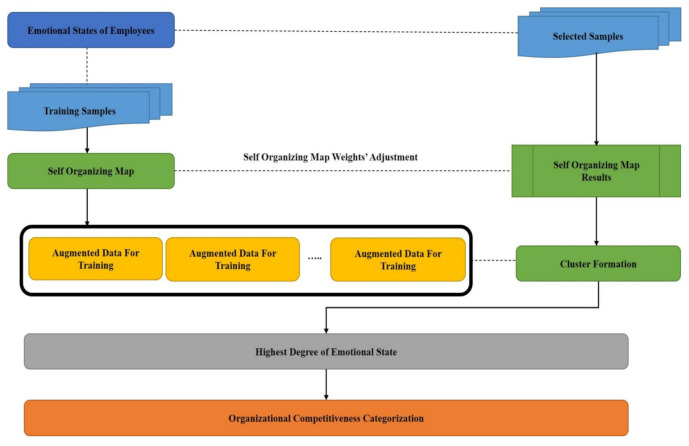
Organizational competitiveness using a self-organizing map.

**Figure 8:**
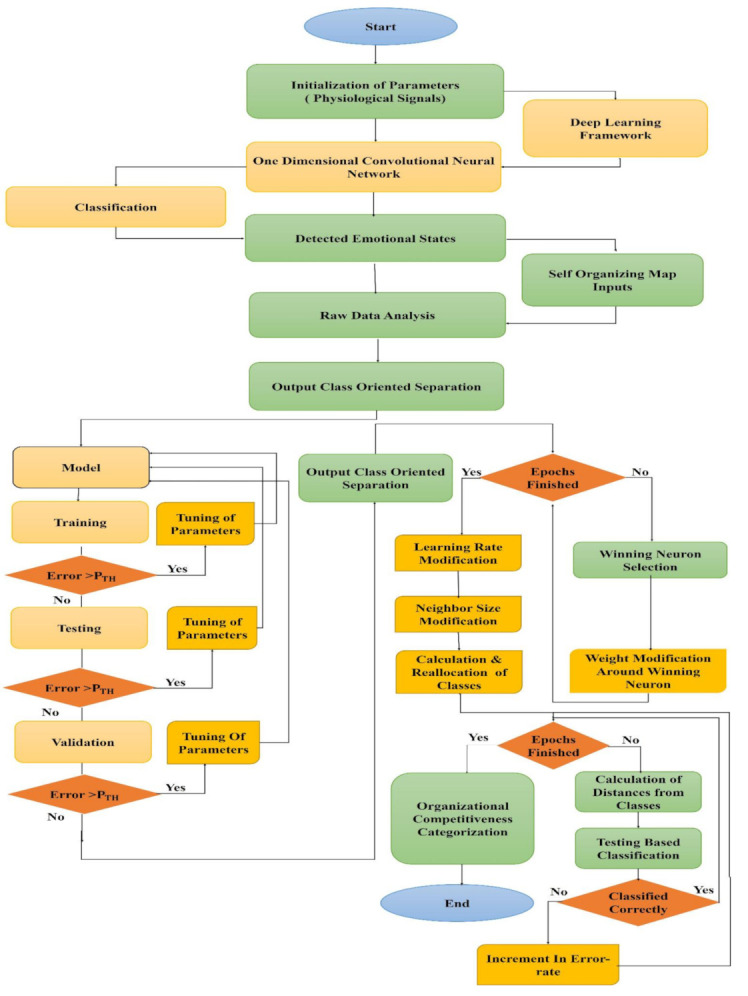
Flow diagram to present the overall process.

Algorithm 2 and Figure 7 demonstrated the clustering procedure for the organizational competitiveness identification through emotional states categorization and Figure 8 summarizes this hybrid procedure containing the ODCNN and SOM and displays the overall flowchart of the proposed work.
**Algorithm 2. Categorization of organizational competitiveness based on the self-organizing map****
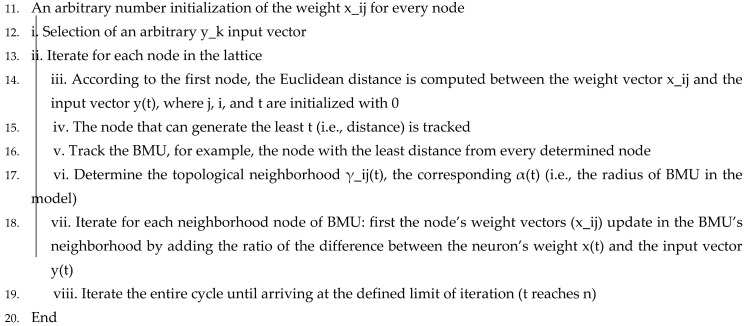
**

## 3. Experimental Results

This study included 1200 participants working in 10 different organizations. The mean age of the participants was 44.20 years, and the majority (52.2%) were male, as listed in Table 1.

The results of the analysis of variance (ANOVA) test indicate no significant difference in BMI values with participants exhibiting different emotional states; however, a significant mean difference was observed for age, BVP, GSR, SKT, valence, and arousal values (Table 2). Participants in a higher age group tend to be happier or more neutral, but the mean age of the participants was lower (39.72 ± 14.38) in the anger group. A higher rate of BVP was observed in a neutral state, followed by happy, excited, and angry. The maximum values were observed in participants in the excited state (8.15 ± 0.70) followed by happy, angry, and neutral for the GSR. For SKT, the lowest mean was observed in the excited state. The lowest values were observed for valence in participants with angry emotions (2.45 ± 0.82), and the highest values were noted when they were excited (9.28 ± 0.23). Furthermore, for arousal, the lowest value was in a neutral state (5.29 ± 0.44), and the most elevated value occurred in an angry state (7.41 ± 0.85).

We also correlated the included parameters with each other in all four emotional states (Table 3). In the angry state, arousal is negatively correlated with valence (*r* = −0.53, *p* < 0.01) and SKT (*r* = −0.49, *p* < 0.01). A negative correlation was also observed in valence and BVP (*r* = −0.14, *p* = 0.01) and SKT and BVP (*r* = −0.23, *p* < 0.001). However, a positive correlation was observed with SKT and GSR (*r* = 0.34, *p* = 0.01). In participants with a happy emotion, valence is positively correlated with arousal (*r* = 0.62, *p* < 0.01) and GSR (*r* = 0.16, *p* = 0.003), and negatively correlated with SKT (*r* = −0.42, *p* = 0.006). In the excited state, arousal and valence are positively correlated (*r* = 0.35, *p* < 0.001), and SKT also exhibited a positive correlation with BVP (*r* = 0.18, *p* = 0.01). In the neutral state, a positive correlation was observed for arousal and SKT (*r* = 0.11, *p* = 0.04), valence and BVP (*r* = 0.16, *p* = 0.003), valence and SKT (*r* = 0.19, *p* = 0.001), and SKT and GSR (*r* = 0.94, *p* = 0.01).

**Figure 9:**
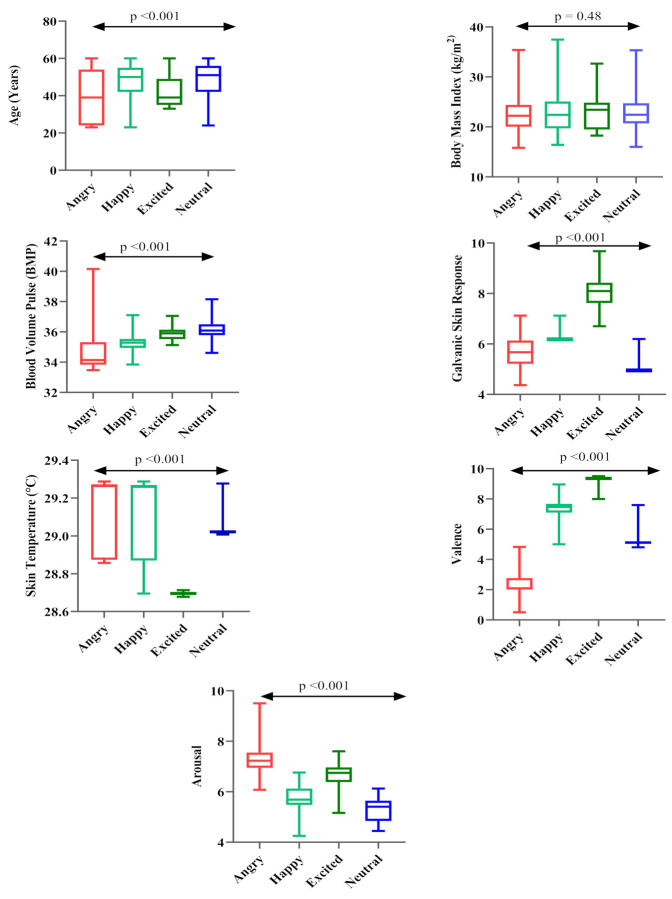
Comparative analysis of identified parameters during different emotional states.

The ANOVA results indicate no mean difference in the BMI of the participants exhibiting different emotional states, though a significant mean difference was observed for age, BVP, GSR, SKT, arousal, and valence. The results of ANOVA are presented in Figure 9.

### 3.1. Emotional State Detection

#### 3.1.1. One-Dimensional Convolutional Neural Network

The ODCNN scheme is employed to classify emotional states. The emphasis is on compact ODCNNs with very few hidden layers or neurons and their implementation in emotion detection for organizational competitiveness classification. This scheme is under the condition that labeled data are limited or application or device-specific solutions are needed to optimize the identification accuracy. Table 4 provides an optimized characterization of the ODCNN for classifying emotional states. The acquired accuracy rate is 99.8%. The total misclassification was recorded as 12, the prediction speed was around 120 observations/s, and the training time recorded was about 1.8652 s (Table 4). In comparison, we analyzed the performance of the two most commonly used and famous algorithms against multiclass classification (i.e., the support vector machine (SVM) and ensemble algorithms).

#### 3.1.2. Support Vector Machine

The fine classification of the input parameters is performed using the SVM. Table 5 provides the SVM characterization and indicates that a precision rate of 83.5% is accomplished. The Gaussian kernel is used with a 2.5 scaling rate and a one-to-many criterion.

#### 3.1.3. Ensemble RUSBoosted Tree (ERT)

The ensemble of primary decision trees with the RUSBoosted (ERT) approach is employed here, which might provide a more precise rate (based on the data). Table 6 provides the ERT particulars, including a precision rate of 71.4%.

**Figure 10:**
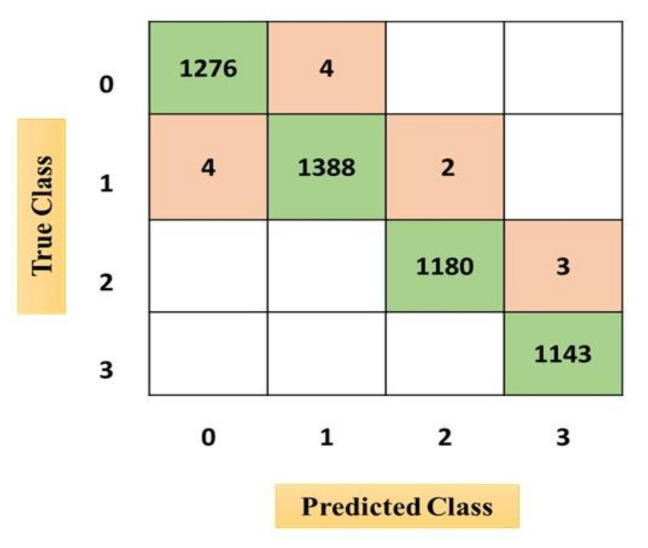
Confusion matrix for the one-dimensional convolutional neural network.

**Figure 11:**
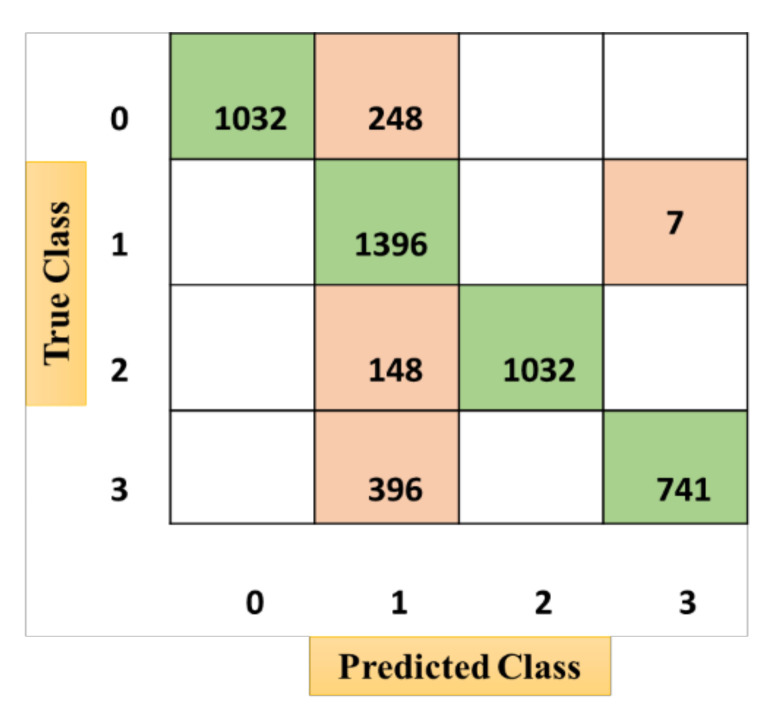
Confusion matrix for the support vector machine.

**Figure 12:**
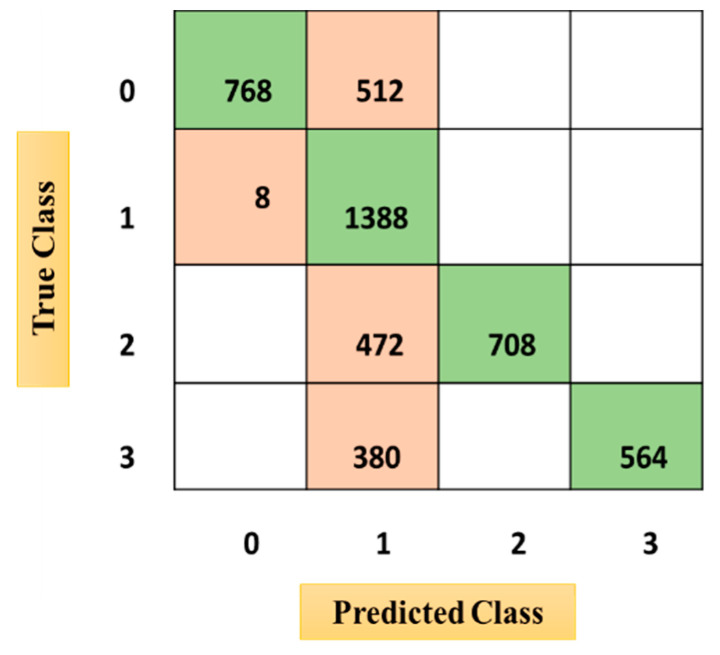
Confusion matrix for the ensemble RUSBoosted tree.

The confusion matrices from Figure 10, Figure 11 and Figure 12 were acquired after performing the emotional state classification using the identified input parameters. There were 1200 instances in the dataset, comprising neutral (0), happy (1), excited (2), and angry (3) emotional states (physiological signal-oriented) data samples. Data augmentation was performed to obtain a dataset of around 5000. The results from the ODCNN show the highest accuracy rate for happy emotional states (1388 samples with six misclassified cases). The neutral state is in the second rank with 1276 correctly classified samples and four misclassified cases. The excited emotional state is in the third rank with 1180 correctly classified cases and three misclassified cases. Finally, the angry emotional state is in the fourth rank with 1143 correctly classified cases and zero misclassified cases. Similarly, the SVM acquired the highest accuracy rate for the happy emotional state with 1396 correctly classified cases and seven misclassified samples, but the overall misclassification rate was higher than for the ODCNN. Likewise, in the ERT, the highest accuracy rate was achieved for the happy emotional state with 1388 correctly classified cases and eight misclassified cases, but the overall misclassification rate was even higher than for the ODCNN and SVM.

**Figure 13:**
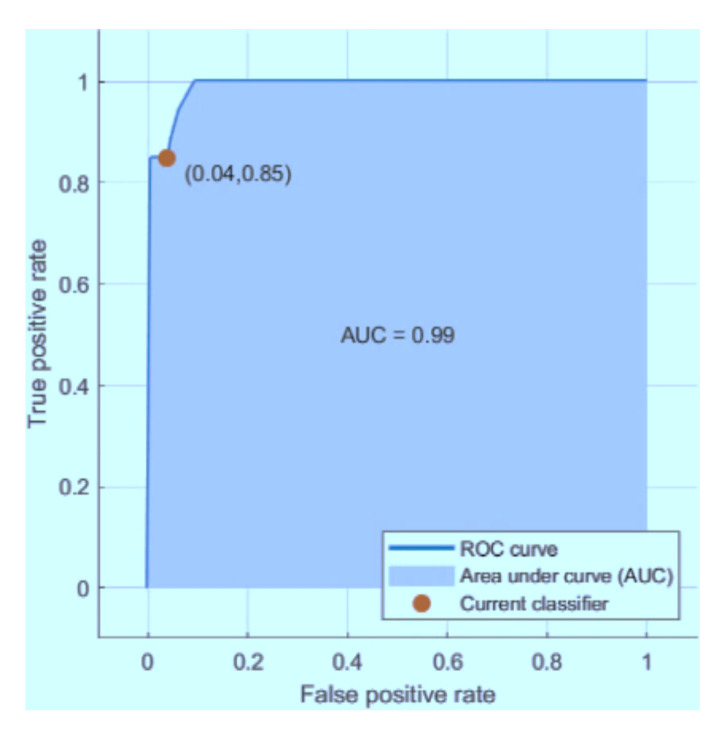
Receiver operating characteristic (ROC) curve for the one-dimensional convolutional neural network.

**Figure 14:**
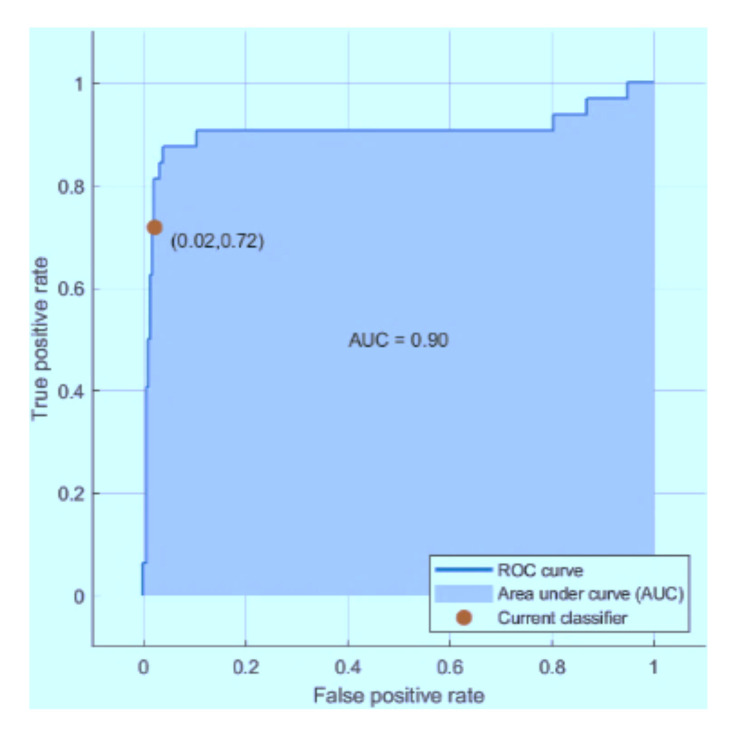
Receiver operating characteristic (ROC) curve for the support vector machine.

**Figure 15:**
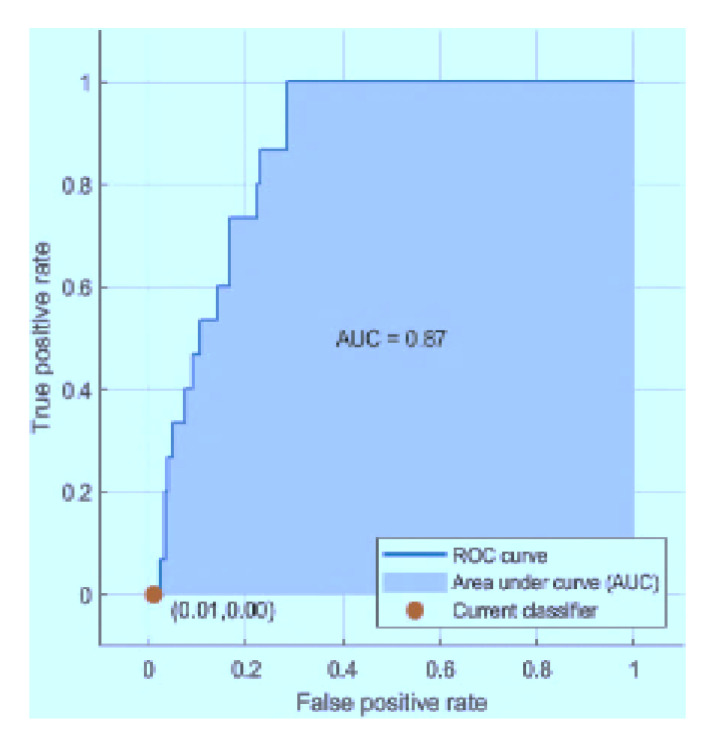
Receiver operating characteristic (ROC) curve for the ensemble RUSBoosted tree.

The graphs below in Figure 13, Figure 14 and Figure 15 represent the receiver operating characteristic (ROC) curves for the ODCNN, SVM, and ERT. For multivariate analysis, the ROC curves are provided. The *x*-axis indicates the (1-specificity) and the *y*-axis in the ROC curves shows the sensitivity for the four output classes. The area under the curve is 0.99 for the ODCNN, 0.90 for the SVM, and 0.87 for ERT.

**Figure 16:**
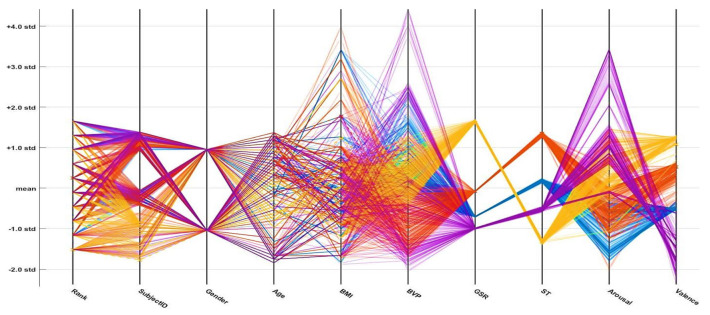
Parallel coordinate plot for the one-dimensional convolutional neural network.

**Figure 17:**
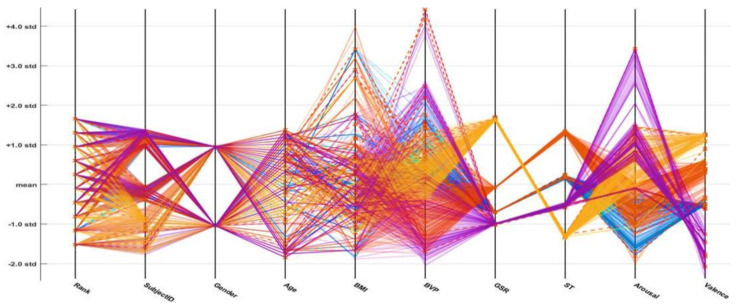
Parallel coordinate plot for the support vector machine.

**Figure 18:**
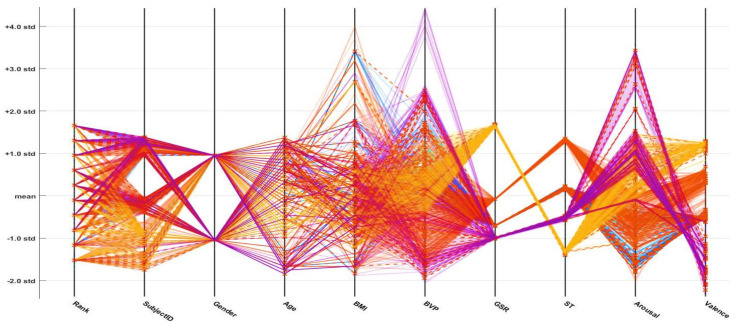
Parallel coordinate plot for the ensemble RUSBoosted tree.

In the following parallel coordinate plots (PCPs; Figure 16, Figure 17 and Figure 18), distinct parameters are plotted corresponding to each other (with the pivot). Each pivot has a substitute scale, as each feature works as a different measurement unit. In addition, each axis can be standardized, keeping every scale uniform. These parameter values are shown as the assortment of lines (they are connected through the axis). Thus, on every pivot, a sequence of points forms a line (entirely connected). The connection between different dataset parameters is presented below the PCPs (based on the standard deviation and mean values). An axis is provided for every parameter (i.e., age, gender, height, weight, etc.), and the axes are parallel to one another. An alternate scale is provided for each axis, as every input parameter works as an alternate unit to classify the emotional state. A progression of lines is used to plot the values associated across each axis. On every axis, a series of points, which are all associated, is represented by each line. The misclassifications are represented by the cross symbol (*x*), and correct classifications are indicated by the dot symbol (.). The PCP demonstrates that effective variables or features of the selected emotions (neutral, happy, excited, and angry) contribute to their discrimination. The plots demonstrate that the ODCNN technique more accurately classifies the defined features into the labeled emotional categories than the SVM and ERT techniques.

### 3.2. One-Dimensional Convolutional Neural Network Structure Optimization

Various hyperparameters, such as network depth, kernel size, and learning rate, were examined to optimize the performance of the chosen technique, ODCNN. The cross-validation setup was used to train the model on the training set. The accuracy of each set of hyperparameters in the ODCNN model was used as a performance metric. The input layer contained 13 nodes, and the output layer contained four nodes representing the four classes. Additionally, a convolutional layer and pooling layer were included in the definition of a convolutional group. The number of groups in the ODCNNs indicates the depth of the network.

Three elements were considered when examining the effect of the ODCNN model on the classification performance: network depth, convolutional kernel size, and learning rate. As listed in Table 7, the numbers from 1 to 16 in the first column represent 16 configurations. The second to the fourth columns denote the three mentioned factors. The row in the table corresponds to an instance. For instance, the fifth configuration indicates that the network depth is four, the convolutional kernel size is three, and the learning rate is 0.00005. The seventh column is the accuracy measure, which indicates the classification performance of the model using the given hyperparameters. The eighth and ninth columns represent the prediction speed and training time, respectively.

It is evident from Table 7 that the accuracy measure increases when the network depth increases from 8 to 11. However, the accuracy decreases when the network becomes deeper from 11 to 14, which indicates that too many parameters in the network may cause overfitting and affect the generality. The accuracy measure increases dramatically and then decreases with the increase in kernel size. The highest accuracy measure emerges when the kernel size is seven, and the accuracy measure increases when the learning rate increases from 0.00001 to 0.0005. Therefore, the best combination is configuration 11. Based on the above work, the optimized parameters are listed in Table 4. A new ODCNN model was trained using these hyperparameters. The model’s classification accuracy is 99.80%, demonstrating that this set of hyperparameters is optimal. Additionally, the time taken using the proposed ODCNN framework is 13.8652 s, which is also fast and suitable for real-time applications.

Two traditional approaches (SVM and ERT) were also tested and compared with the intended ODCNN method. All algorithms, written in Python 3.8, were implemented in TensorFlow 1.15 and executed on a laptop with the Nvidia GeForce graphics card, an 8th generation Intel Core i7 processor, and a 1 TB solid-state drive. As mentioned, the emotion classification was achieved using the identified features and the ODCNN classifier. Remarkably, a high accuracy rate of 99.80% was achieved, which is better than the other two methods. The results indicate that the proposed ODCNNs can exploit the latent feature representations of the dataset, and the emotional states can be classified accurately.

### 3.3. Organizational Competitiveness Categorization through the Self-Organizing Map

**Figure 19:**
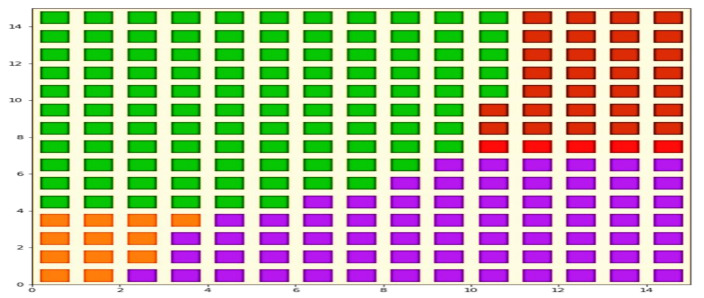
Clustering based on emotions through the self-organizing map.

The output of the SOM, representing the classification of organizational competitiveness based on employee emotions during their mutual interactions in the workplace, is provided in Figure 19. The square cells represent nodes. The K-means approach was applied for partitioning clusters, generating four clusters through this approach. Different colors represent the membership of clusters. A high degree of the angry emotional state indicates low-level organizational competitiveness. In contrast, a high degree of the neutral emotional state represents moderate-level organizational competitiveness. However, a high degree of the happy and excited emotional states in the employees indicates high-level organizational competitiveness. In the following map, a high level of possession of organizations is shown by the green cluster (at the top of the map), representing the happy emotional states in the majority of employees. The map depicts a high degree of organizational competitiveness. A comparative view of the proposed work with a few prior similar types of research is provided in Table 4.

## 4. Discussion

With the successful introduction of AlexNet, the era of deep TDCNNs began. Within a short period, TDCNNs supplanted conventional pattern classification techniques. Deep CNNs have evolved into the primary tool for any deep learning task. Apart from using a deeper network with sparse connections, the central concept is that GoogLeNet achieved the highest object detection results in ImageNet Large Scale Visual Recognition Challenge 2014 using an ensemble of six CNNs. Since then, deep CNNs have grown in popularity, eventually becoming the industry standard for various identification and classification applications. Additionally, they have been widely applied to the processing of sequential data, such as natural language processing and speech recognition, and 1D signals, such as vibration [42,43,44].

Apart from the superior performance that deep CNNs accomplish, another prominent feature is that, unlike standard ANNs, they can incorporate feature extraction and classification tasks into a single body. In contrast, conventional machine learning techniques typically involve preprocessing and rely on stabilized and indicative features, which are inefficient and take a considerable computational load. The CNN-based methods learn to extract optimized features directly from the problem data to maximize classification accuracy. This outcome is the key element for significantly improving classification performance, which is why CNNs are appealing for complex applications. However, standard machine learning strategies have remained unchecked for 1D signals, as deep CNNs were designed and developed solely for 2D signals. The application of these strategies to 1D signals is not concise, particularly when data are abundant. Additionally, the direct use of a deep CNN for 1D signal processing applications necessitates an appropriate 1D to 2D conversion [44,45].

The ODCNN is used in this study because deep CNNs have several disadvantages and limitations. Primarily, it has been well established that CNNs have high computational complexity, necessitating specialized hardware designed specifically for training. Thus, TDCNNs are unsuitable for real-time applications on mobile and low-power/low-memory devices. Additionally, proper training of deep CNNs requires a large training dataset to maintain a reasonable degree of generalization capability. It may not be a feasible option for numerous practical 1D applications in which labeled data are limited. Kiranyaz et al. proposed the first modular and resilient ODCNNs capable of operating explicitly on patient-specific ECG data in 2015 to address these shortcomings [46].

Moreover, ODCNNs have gained popularity in a relatively short period due to their superior performance in a wide range of signal processing applications, including early arrhythmia diagnosis in ECG beats [45,47] and monitoring and detecting underlying health problems [48,49,50,51,52]. However, deep ODCNN approaches have recently been proposed for anomaly detection in ECG signals. These deep configurations share many of the same disadvantages as their 2D peers [53,54,55]. Numerous techniques have been used to enhance the generalization performance of the deep ODCNN, including data augmentation, batch normalization, dropout, and majority voting [56]. Regularization is critical, especially in ill-posed problems with insufficient data. Regularization can be interpreted as a technique for achieving algebraic stability during the reconstruction process but is much more than a simple stabilization technique [57,58]. Data augmentation is a prevalent method for regularizing data. The scheme’s core premise is to augment the training dataset via transformations that reduce overfitting. The experimental results reveal that data augmentation makes the classification process more efficient.

Recently, with deep learning-oriented advancements, research has been conducted on emotional state classification. Previously proposed emotion detection approaches are usually dependent on EEG, ECG, facial, or vocal cues and are difficult to compute in regular day-to-day life. Generally, these approaches are challenging to rely on and use because the outcomes are distinctive and rely on the domain specialist or dataset. Physiological cues have the benefit of the option to be estimated noninvasively in regular daily life. Physiological signal-oriented gadgets are now commonly employed. Additionally, applications using physiological cues are primarily effectively developed and employed. Following this, independent deep learning-oriented frameworks limit preprocessing approaches, such as noise filtration, feature extraction [59], and others. Different organizations have established these frameworks for EC in different domains for diverse purposes.

We presented an experimental study to recognize human emotions in response to mutual interaction using preprocessed demographics (gender, age, and BMI) and physiological signal-based datasets (BVP, GSR, SKT, valence, and arousal). From the experimental results, we observed that physiological signals, such as BVP, GSR, SKT, valence, and arousal, could be used to classify human emotions more precisely than features based on the EEG and ECG. Moreover, the accuracy of emotion recognition in response to mutual interaction increases by fusing the modalities mentioned earlier. Our proposed (ODCNN) emotion recognition schemes are compared with state-of-the-art techniques in terms of accuracy, as presented in Table 8. To verify the algorithm, we conduct extensive experiments to demonstrate that the algorithm improves the generalizability of the ODCNN through optimization and is robust to the choice of hyperparameters, as listed in Table 7.

The ODCNN, SVM and, ERT classifiers were used for classification. The results reflect that the ODCNN classifier outperformed SVM and ERT in this research. The combinations of the features were input into the three classifiers. The ODCNN has a better classification rate, and the complex network features are effective in recognizing emotion. The study provides a new research idea in emotion recognition. The emotion recognition problems in the references of Table 8 are all multiclassification. With the proposed classifiers (ODCNN, SVM, and ERT), the achieved accuracy is 99.8%, 83.5%, and 71.4%, respectively, for the emotional states of neutral, happy, excited, and angry. According to Li et al. [5], a brain–computer interface-based emotion recognition scheme with an improved particle swarm optimization for feature selection was employed with an accuracy of 95%. Graterol et al. [60] proposed a method for emotion recognition and achieved an accuracy of around 53% for classification tasks. Seo et al. [21] used machine learning methods for boredom classification using EEG and GSR data to reach an accuracy of 89%. An EEG-based emotion classification study using a long short-term memory network with an attention mechanism was proposed in [61]. The experimental results demonstrated that the classification accuracy was around 99% for emotion classification.

Due to these advancements, emotional state detection or classification is a vast and robust approach for correspondence with medically introverted persons who cannot communicate their feelings without help from others. Moreover, in the treatment of mental health and brain–computer interaction, perceived emotional state detection systems could be employed. Through this research, experimentation on emotional state detection was conducted (generally with limited preprocessing steps to ensure the emotion detection precision rate) on how this emotional state of organizational employees affects the overall degree of competitiveness of the organization. Likewise, this work supports multiple degrees of emotional states and monitors the changes in emotional states over time to improve the precision rate of emotion detection. A hybrid approach was used for this work: one (i.e., ODCNN) for classifying the emotional states of employees and a second (i.e., SOM) for detecting employee emotion-oriented organizational competitiveness.

Moreover, the current model was checked and explored using a preprocessed physiological dataset. Gathering physiological signals or using an alternate kind of dataset requires ensuring identical performance. For future directions in research, input data (unprocessed and well-processed data) must be compared according to different environmental conditions.

## 5. Conclusions and Future Work

In this manuscript, a deep learning-oriented ODCNN approach is employed to classify the emotional state of organizational employees based on a preprocessed dataset obtained using physiological signals, which has acquired popularity as a reasonable approach (in the domain of EC). Second, the presented approach classifies organizational competitiveness (using a SOM) by clustering the emotional states of organizational employees. The outcomes depict a 99.8% precision rate individually, using a two-stage classification procedure for employee emotion classification and categorizing organizational competitiveness. For the proposed experimentation, organizational competitiveness is classified using the combined working of ODCNN and SOM. We followed with the research results that physiological signals such as BVP, GSR, SKT, valence, and arousal could be used to more concisely characterize human emotions than EEG and ECG characteristics. Moreover, aging, especially experience, along with the emotional detachment and balance that it brings, is an indispensable resource in the professional world.

For future directions, the imbalance in the dataset must be addressed (using the regularization approach). It can more efficiently cater to the physiological signals and other kinds of signals that are crucial in the precise prediction of emotional states. Further improvements in time consumption (while performing the emotion state classification and categorizing organizational competitiveness) are also vital, using other physiological signs and datasets. Along these lines, besides collecting data from various sources for the proposed work, the daily life (real-time) feasibility must be assessed. Moreover, the comparison of different inputs can be made using different sources (e.g., unprocessed or well-processed data), and noise injection can be performed in different physiological signals (in the middle layers of a model). Later, further research can be conducted on brain activation, according to the transformation and fluctuation in the physiological signs and cues. How do these variations affect a person’s emotional states and, consequently, what is the influence on the overall effect toward organizational competitiveness and effectiveness?

## Figures and Tables

**Table 1:** Sociodemographic data of the participants.

Age (Years)	Mean ± Standard Deviation	44.20 ± 11.50
Gender	Female	574 (47.8%)
	Male	626 (52.2%)
Organizational Ranking	Rank 1	130 (10.8%)
	Rank 2	131 (10.9%)
	Rank 3	121 (10.1%)
	Rank 4	127 (10.6%)
	Rank 5	123 (10.3%)
	Rank 6	135 (11.3%)
	Rank 7	100 (8.3%)
	Rank 8	125 (10.4%)
	Rank 9	110 (9.2%)
	Rank 10	98 (8.2%)

**Table 2:** Comparative analysis of variables in different emotional states.

Emotional Status (*n*, %)	Age	Body Mass Index	Blood Volume Pulse	Galvanic Skin Response	Skin Temperature	Valence	Arousal
Angry (236, 19.7%)	39.72 ± 14.38	22.50 ± 3.50	34.81 ± 1.43	5.67 ± 0.67	29.14 ± 0.18	2.45 ± 0.82	7.41 ± 0.85
Happy (349, 29.1%)	46.26 ± 11.50	22.94 ± 4.27	35.30 ± 0.69	6.19 ± 0.05	29.08 ± 0.20	7.28 ± 0.60	5.74 ± 0.41
Excited (295, 24.6%)	41.88 ± 7.84	22.64 ± 3.14	35.88 ± 0.42	8.15 ± 0.70	28.69 ± 0.01	9.28 ± 0.23	6.71 ± 0.45
Neutral (320, 26.7%)	47.40 ± 10.47	22.75 ± 3.68	36.19 ± 0.61	4.95 ± 0.07	29.02 ± 0.01	5.38 ± 0.72	5.29 ± 0.44
*p*-value	<0.001	0.48	0.001	<0.001	0.001	<0.001	<0.001

**Table 3:** Correlation analysis in different emotional states.

Correlation Analysis in the Angry Status							
		Age	Arousal	Valence	Skin Temperature	Galvanic Skin Response	Blood Volume Pulse
Body Mass Index	PC	0.49	−0.09	0.09	0.12	0.03	−0.12
	*p*-value	<0.001 *	0.15	0.16	0.07	0.64	0.06
Blood Volume Pulse	PC	−0.08	0.08	−0.14	−0.23	0.07	
	*p*-value	0.20	0.20	0.01 *	<0.001	0.30	
Galvanic Skin Response	PC	−0.05	−0.05	−0.06	0.34		
	*p*-value	0.43	0.41	0.25	0.01 *		
Skin Temperature	PC	0.13	−0.49	0.10			
	*p*-value	0.05	<0.001 *	0.09			
Valence	PC	0.14	−0.53				
	*p*-value	0.02 *	<0.001 *				
Arousal	PC	−0.09					
	*p*-value	0.15					
Correlation Analysis in the Happy Status							
		Age	Arousal	Valence	Skin Temperature	Galvanic Skin Response	Blood Volume
Body Mass Index	PC	0.28	0.04	−0.04	−0.06	0.06	0.01
	*p*-value	<0.001 *	0.44	0.36	0.77	0.25	0.83
Blood Volume Pulse	PC	0.06	−0.17 *	0.16	0.03	0.03	
	*p*-value	0.26	0.001 *	0.07	0.54	0.61	
Galvanic Skin Response	PC	0.01	0.09	0.16	−0.19		
	*p*-value	0.99	0.11	0.09	0.07		
Skin Temperature	PC	0.03	−0.08	−0.42 *			
	*p*-value	0.96	0.10	0.006			
Valence	PC	−0.06	0.62 *				
	*p*-value	0.20	<0.001 *				
Arousal	PC	−0.04					
	*p*-value	0.45					
Correlation Analysis in the Excited Status							
		Age	Arousal	Valence	Skin Temperature	Galvanic Skin Response	Blood Volume Pulse
Body Mass Index	PC	0.19	0.13	0.05	0.06	0.02	−0.08
	*p*-value	<0.001 *	0.09	0.34	0.28	0.71	0.15
Blood Volume Pulse	PC	0.01	−0.30	−0.10	0.18 *	0.02	
	*p*-value	0.88	0.61	0.08	0.01 *	0.99	
Galvanic Skin Response	PC	−0.04	0.08	0.06	0.01		
	*p*-value	0.44	0.13	0.28	0.86		
Skin Temperature	PC	−0.03	−0.36	−0.03			
	*p*-value	0.50	0.54	0.51			
Valence	PC	0.01	0.35				
	*p*-value	0.82	<0.001 *				
Arousal	PC	0.15					
	*p*-value	0.01 *					
Correlation Analysis in the Neutral Status							
		Age	Arousal	Valence	Skin Temperature	Galvanic Skin Response	Blood Volume Pulse
Body Mass Index	PC	0.24	0.01	−0.13	0.09	0.10	−0.08
	*p*-value	<0.01 *	0.74	0.18	0.10	0.06	0.14
Blood Volume Pulse	PC	−0.14	−0.005	−0.006	−0.14	−0.14	
	*p*-value	0.01 *	0.933	0.91	0.08	0.01 *	
Galvanic Skin Response	PC	0.04	0.09	0.16	0.94		
	*p*-value	0.43	0.08	0.003 *	<0.001 *		
Skin Temperature	PC	0.04	0.11	0.19			
	*p*-value	0.41	0.04*	0.001 *			
Valence	PC	−0.11	0.02				
	*p*-value	0.03 *	0.72				
Arousal	PC	−0.01					
	*p*-value	0.89					

PC: Pearson correlation; * significant *p*-value.

**Table 4:** One-dimensional convolutional neural network characterization.

Parameter	Value
Accuracy Rate	99.8%
Total Misclassification Cost	12
Prediction Speed	~420 obs/s
Training Time	13.8652 s
Model Type	ODCNN

**Table 5:** Support vector machine characterization.

Parameter	Value
Accuracy	83.5%
Total Misclassification Cost	802
Prediction Speed	~1201 obs/s
Training Time	11.8658 s
Model Type	Gaussian SVM
Kernel Function	Gaussian Kernel
Kernel Scale	2.5
Multiclass Method	One to Many

**Table 6:** Ensemble RUSBoosted tree characterization.

Parameter	Value
Accuracy	71.4%
Total Misclassification Cost	1398
Prediction Speed	~881 obs/s
Training Time	14.5868 s
Model Type	Boosted Trees
Ensemble Method	RUSBoosted
Learner Type	Decision Tree
Maximum Number of Splits	30
Number of Learners	20

**Table 7:** Comparison of the configuration results.

Configuration Number	Network Depth	Kernal Size	Learning Rate	Activation Function	Model Type	Accuracy (%)	Prediction Speed (obs/s)	Training Time (s)
1	3	3	0.00005	ELU	ODCNN	95.53	~180	3.8862
2	3	5	0.00001	ELU	ODCNN	97.74	~204	4.2252
3	3	7	0.0005	ELU	ODCNN	92.92	~230	4.3711
4	3	9	0.0001	ELU	ODCNN	90.03	~308	6.1201
5	4	3	0.00005	ELU	ODCNN	91.47	~198	6.0529
6	4	5	0.00001	ELU	ODCNN	97.13	~276	9.8658
7	4	7	0.0005	ELU	ODCNN	96.22	~332	11.2355
8	4	9	0.0001	ELU	ODCNN	97.93	~412	13.1341
9	5	3	0.00005	ELU	ODCNN	98.79	~208	8.3424
10	5	5	0.00001	ELU	ODCNN	99.56	~302	11.0012
11	5	7	0.0005	ELU	ODCNN	99.80	~420	13.8652
12	5	9	0.0001	ELU	ODCNN	96.53	~538	15.4654
13	6	3	0.00005	ELU	ODCNN	93.76	~272	10.2232
14	6	5	0.00001	ELU	ODCNN	92.23	~399	12.1113
15	6	7	0.0005	ELU	ODCNN	95.02	~454	20.7345
16	6	9	0.0001	ELU	ODCNN	91.88	~688	22.8952

**Table 8:** Comparison with previous research.

Research	Accuracy
[5]	95%
[21]	89.29%
[60]	53%
[61]	99.01%
[62,63]	97%
-------------------------------------------------------------------------------------------------	
Proposed (ODCNN)	99.8%
SVM	83.5%
ERT	71.4%

## Data Availability

Data will be available on request.
